# Constructing Crown-And-Bridge-Work

**Published:** 1892-09

**Authors:** G. W. Melotte

**Affiliations:** Ithaca, N. Y.


ARTICLE IV.

### CONSTRUCTING CROWN-AND-BRIDGE-WORK.

BY DR. G. W. MELOTTE, ITHACA, N. Y.

Gold in Holy Writ stands for truth, and of all metals that man has been able to manipulate, I think it stands at the head. Platinum ranks by the side of gold, and in the construction of crown- and bridge-work we have to deal with gold and platinum.

It is curious to note the effect that can be produced by placing a piece of pure gold on a piece of platinum and passing it through the rolls together, first heating them over a Bunsen burner. You find, if the surfaces are clean, there will be perfect welding, as perfect as you can weld two pieces of cohesive gold foil. This form of gold and platinum is used in backing up teeth, using pure gold to bring the tooth that is too white up to a richness in color that will serve the purpose. Another curious fact is, that pure gold under the blow pipe, 18 carat—and I do not know but any carat gold—can be readily welded. The surface must be cleansed by applying borax rubbed on a slate or piece of ground glass. I do not find it necessary in welding gold bands to put them into sulphuric acid, the borax answers every purpose of purifying the surface. To weld a gold band let the edges lap, bringing the edges into perfect co-aptation, apply the creamy borax to the parts to be united, then with a flowing or soft flame of the blow-pipe bring the gold to a perfect redness when a concentration of the flame will cause a slight fusion of the surfaces and thereby welding.

I learned this method of welding gold from Dr. Bing, of Paris, a year ago last summer. I saw Dr. Bing do the work nicely, and thought it would be a practical thing with me. Finding I could not do it very well at first, I laid it aside until Dr. Templeton visited me last August, and I showed him this method of welding coined gold. He seemed to be very much pleased with it, and was able to do it himself, and that gave me great encouragement, and so from that time to the present I have welded all my bands. I use no solder except soldering on the caps in forming cusps. I do not like to use very much gold in the anterior portion of the mouth. By beating the gold after a band is formed and doubling it over, you can proximate the shape of a cuspid, and it is easy to weld. I have been able in one case to join a piece of gold on by making the upper edges free, then by careful manipulation of the blow-pipe I was able to add one piece after another till I made a cuspid with a thick end or point over the surface. I think this method of uniting bands much better than to use solder. Soldering produces weakness of the band when it unites at a slight heat. If No. 18 carat gold, it lowers the character of the gold in the main at that point. If the band should require stretching, it may be done with a hammer at the point where the surfaces have been united in the weld without reducing the general thickness of the band.

We will now construct the anchorages for a bridge. The case is one involving the loss of the first and second bicuspid, together with the first molar, right side, lower jaw, anteriorly we have a sound cuspid tooth and a firm laternal incisor root; posteriorly the molar is sound, with no antagonizing tooth in the upper jaw. We will prepare the lateral root for a Richmond crown, the construction of which I presume you are all familiar. The cuspid may be banded by excising the crown. A tooth cut off and nerve pulp destroyed, banded and capped in the style of a Richmond crown with porcelain base, gives stronger anchorage for a base than that of a banded cuspid tooth, or, in fact, any tooth banded. It is true, we have the prejudice of patients to contend with, and possibly our own timidity in cutting of teeth. I am not afraid to cut off sound teeth, and think it would promise greater strength and permanency to do so.

But in this case the sound cuspid has been banded, the lateral root furnished with a Richmond crown, completing the two anterior abutments. We will now prepare the second molar for the posterior anchorage. In this case there is no occluding tooth in the upper jaw. We all know it is important that the bulbous portion of a tooth should be removed and straightened, so that the band when fitted may pass down under the free margin of the gum, and you can crowd it down; you had better have it go beyond the point your judgment would seem to indicate rather than to have it too short. I think it is one of the sources of protecting teeth from decay. We will say, we have taken off the bulbous portion of the tooth. To obtund sensibility, it is well to add alcohol to water; it will also sharpen the corundum, so that it will cut more readily than otherwise, and, of course, you all know a sharp knife is better than a dull one.

Now, we have prepared the tooth with the exception of removing the cusps. In this case I said there was no occluding tooth. It stood alone, without any antagonism, and we are going to benefit the tooth by giving it something to do. In making my bands, I wrap a piece of gold around the tooth, lap it, hold it with the fingers, and mark it. I think most crown workers who do a great deal of bridge-work fall into the habit of doing it in this way without measurements. It is well enought for a man to measure when he first commenccs,

Now, we have the size, and this (illustrating) will represent the gold band. It have got it lapped at the point you see. If you use solder instead of welding in the commencement, it will perhaps be well enough to take a little whiting mixed in alcohol and coat the surface, leaving a point just at the top, joining with soldering there. This leaves the bottom of the band open. If you drive it on the tooth there will be less resistance, and you can fit a band to a tooth easier than if you had joined it from the top to the bottom or the whole length. You fill in the end with plaster of Paris mixed with sulphate of potash or salt and water, so that it will set quickly and fit the band in there, tamp it down so the air will bubble out. As soon as it sets carefully remove the band. The portion of the band not occupied by plaster of Paris is filled with fusible metal melting at 150° F. You cut it off with excising forceps and drop it into the band, then with a puff of the blow-pipe melt it. The plaster is now inverted. You have placed your pieces of fusible metal into the band, and, with a puff of the blow-pipe, have melted them. Do not overheat, but barely melt the pieces of metal, and fill it a little more than full. As soon as it is congealed and hard, remove the plaster. You can trim gold down within a line or line and a half above the cusps, and then using 22 carat gold there will be no trouble with the hammer and burnishers to burnish it over into the cusps. You can take an impression in plaster and make a die and counter die with fusible metal melting at 212°.

After you burnish gold down into this fusible metal or cusps you make a die, swage out a piece, then with a small ladle placed in boiling water the metal will melt below the temperature of the water. It is better to do it in that way than to resort to the blow-pipe. I find in using the blow-pipe, that there is great danger of overheating, and the pieces of metal unite with gold and spoil it. Let me say here, by way of caution, if you try this method of fusible metal, and are not careful to remove every particle of it before you put your pieces under the blow-pipe again, you will wish you had never seen Melotte or anybody else. There would be a union of the fusible metal with the gold, destroying the integrity of the gold. If you are careful in the operation to remove the diffusible metal perfectly, you will have no trouble in soldering on the little cap of gold into the gold band. Now, you have made the crown with the exception of the band, contouring and fitting it to the tooth in the mouth, and you have taken an impression, you have done the whole work of making the crown, getting a crown that will go on and answer the purpose of the posterior abutments nicely.

A Member: Do you band before you put the fusible metal in?

Dr. Melotte: I do not. It would be well to do it, but I have no time, as a rule. to do that. Perhaps it would be well for a man to do it when he first commences to do this kind of work.

A Member: Will you please tell us how you weld it?

Dr. Melotte: "The Lord breathed into man the breath of life." I use some of it in connection with the blow-pipe, and by coating the surface with borax, you are enabled to bring it up to the heat of fusion. The molecules of the upper layer to the lower layer will come in contact with heat and readily unite. If you are not careful, you will spoil your band; so it is well enough to experiment with pieces of gold in soldering before adapting it in actual practice. In doing this work, it is well to have a blacksmith's heat, which is a general heat, heating your band up thoroughly before you concentrate the heat, otherwise it is better, perhaps, to point the flame a bit, heating it up until the material is ready to melt; then you get your welding. You may spoil a number of bands of gold before you are able to do the actual work, but you will be surprised how well you can do it if you learn to emit a nice flame with the blow-pipe.

A Member: Why do you use shellac on porcelain?

Dr. Melotte: Because in burning it carbonates and gives you a layer of charcoal next to the porcelain. It protects the porcelain more perfectly than it is protected with the ordinary investment of plaster of Paris.

Dr. Harroun: I am in the habit of coating the surface of a tooth, to prevent the plaster of Paris from melting. It forms a coating there, so to speak, prevents the plaster from shrinking down, and forms a roughness on the surface.

Dr. Melotte: That point was suggested to me by a dentist of Utica, in combining the porcelain and gold. He told me that was the course he pursued. I find it a nice thing to do. I am conscious of the fact that with porcelains you cannot be too careful to keep them from contact, Considerable expansion and contraction take place when the molecules are under the excitement of returning to their primitive condition. Of course, there is great danger of breaking. Dr. Starr suggested that pieces of mica be put between the porcelains we use. In olden times it was suggested, that a piece of tissue paper or writing paper put in between would prevent breaking. I think mica is a very good thing, indeed, for this purpose. We should see that the porcelains are not in contact when they are invested.

I have reached a point now where I have constructed my abutments. I call your attention to one of the most important points connected with the work, and yet one that is simple—taking the impression. If this tooth (illustrating) leans toward the anterior teeth, or if there is a wedge-shape or straight space, you may have trouble from breaking of the plaster, making your impression imperfect. To obviate that, I have a piece of modeling compound and press it with my fingers between the teeth. I remove it, put it into cold water, then with a knife I trim on the buccal and lingual surfaces, leaving them a little narrower at the top. Before placing it back, I warm the surface, and direct the patient to bite down on it, leaving an imprint of the occluding teeth. Be careful to see that the patient bites correctly. Now remove the pieces again and trim, so that the modeling compound will come in contact with the anterior and posterior portions of the band, but not to cover any more than you can help. You have trimmed it and replaced it. It is in position. The patient has bitten down on it. You may let them try it again, if you are certain they will bite in the same place. The imprints of the cusps are there. Now you take an impression in plaster, the modeling compound being in position. You take your plaster, and if the compound remains in the mouth and does not give away with the plaster, remove it and put it in its place. The model drawn on the board is but a rough sketch. It represents the two centrals and a lateral together with a cuspid with its band on. Now, in taking an impression, I find that the imprint of this tooth is in the impression. There are few men who practice making impressions with bands on preparatory to making a restoration or bridge; they take impressions in modeling compound. They may be experts in that particular line, and they may succeed in a small piece of bridge work, and may with their method save time, but I would not attempt to do it. A gentleman said to me a short time ago, "It takes so long to remove a plaster impression from the model," and that is one of the reasons why he does not use it. Then, again, sometimes patients object to certain things you want to do. My patients do not object, because I do just as I have a mind to. As a rule, I do, or else I do not do the work.

I have taken the plaster impression, and am about to pour into it plaster of Paris to make my model on which my bridges are constructed. I take two pins and place them into the impression of two of these teeth a little ways (illustrating) apart. The heads are standing up, and the impression is before you, and you are looking down into the imprints of the teeth. I take the pins, place them in the cutting edge of the teeth in this case, then I take some little bits of fusible metal and cut it off with excising forceps and place them into the imprint of the teeth, and with a puff of the blow-pipe fuse the metal, and jar it down same as with plaster, and then you have got the tips of the teeth like that which you see represented on the board. Dr. Templeton has three or four combined tips that I removed from a case while he was with me. He will show these tips to you at my clinic this afternoon. I will take an impression of some one's mouth and show you the method of making these tips. You cut off the ends of the pins and leave the dowels around them, leaving the pins one-half or one quarter of an inch in length, then fill up the model with investment material, plaster of Paris and marble dust. One-third marble dust and two-thirds plaster ot Paris is about my formula. Your plaster is hard, you cut away and down on to these teeth, cutting away tbe plaster impression you cut to the tips. Being metal you are in no danger of marring them. I have got to a place now where I find my modeling compound is in the same position with the cusps upward (this being a lower case) that it was when the patient left the imprint. You readily see that. When I have taken an impression of the occluding teeth (and I take it with plaster), I pour in fusible metal quickly. If I do not get a perfect occlusion, I can pour it several times; I get it quicker than it takes the plaster to set.

The advantage in standing before you and speaking to you in this way is, that I am enabled to command the attention of every one of you at the same time, and am able to state points in the construction of this work which would be very difficult for you to see at a clinic, and it would be likewise difficult for me to perform in the time allotted for a single clinic. I could not do it. There is not a bridge worker in existence (if there is one I would like to see him) who has not had more or less failures, and if he has been at work four or five years in this line, and has not had quite a good many failures, then he must be a better man than I am, and such a man is in danger of toppling over. Our successes flatter us; our failures halt us, and make us consider the ground that we have been over, and make us a little more careful in the future.

A Member: Will you tell us how you make a dummy?

Dr. Melotte: I will state first what happened in my office. My assistant was working for an elderly gentlemen, and he got to the point of adding the dummy, and he turned to me and said, "What shall I do with the dummy?" The man looked up at him with daggers in his eyes, but the young man gave him to understand that he did not mean him.

I do not know how they came to be called dummies, unless they were considered dummies who worked at them first.

I will make the first dummy, and I shall prefer to take an ordinary cuspid tooth, and in grinding it I may find it too long from the pins up to the cutting edge. Dr. Richmond showed me this method of grinding a cuspid tooth to keep the proportions perfect—that is, to grind from the lingual toward the labial surface, as perhaps you are able to see this tooth (illustrating). You take a tooth that is blunt on the end, the advantage is that you smoke it upon the porcelain, so that when you add gold you have a better looking tooth than you would otherwise have; then, besides, you first put a piece of backing on, and this backing and the tooth are ground on the surface, the backing being beveled with the porcelain. You take a piece of thick gold, and where you want great strength on the cutting edge and desire to prevent the teeth from breaking, I should use gold that has about five per cent. of platinum, using No. 23 or 24 and let it extend about a line above the cutting edge, wax it on when it will be ready for investment. You take a die and swage out a bicuspid, take one of the bicuspid dies, swage out a piece of gold, place it on, bevel toward the outer cusp, so that it will fit on to the tooth in the manner in which I hold this (illustrating), wax it in place and invest it, varnishing it at first. Invest in plaster of Paris, marble dust and sand, then after the investment has set, dry it out, melt out your wax, add your gold carefully, you may put in tips of gold coin or filings, fill it up with filings and solder, and in that way you have got your dummy made for your first bicuspid. Proceed with the next and with the molar in a similar manner, and you have your dummies, which, of course, you have tried on and arranged with a view to the occluding teeth. Of course, this work needs care and time, and I cannot find language to describe to you definitely just how to proceed. There is an important point I want to make, and that is that each one of the dummies should be ground, the porcelain and the gold, so that they will touch the ridge, and especially in those cases where there are any bridges which fail to have touched, because the tissues will kindly form around the points of contact, and you need not be much afraid of inflammation, and if you get a little hypertrophy you can allay it as well with a satured solution of salicylic acid as anything, and after a little the gum will kindly form and it comes up around rather than shrinks away from the dummy.—*Ohio Dental Journal.*

